# Structural dynamics of sphingosine kinase 1 regulation and inhibition

**DOI:** 10.21203/rs.3.rs-6575060/v1

**Published:** 2025-05-07

**Authors:** Baharak Abd Emami, Ahmed Shubbar, Hope Woods, Mahmoud Moradi, Reza Dastvan

**Affiliations:** 1 Department of Biochemistry and Molecular Biology, Saint Louis University School of Medicine, St. Louis, MO 63104, USA; 2 Department of Chemistry and Biochemistry, University of Arkansas, Fayetteville, AR 72701, USA.

## Abstract

Sphingosine kinase 1 (SK1) produces sphingosine-1-phosphate, a bioactive lipid implicated in cancer progression and other diseases. Despite its clinical relevance, the structural and dynamic basis of SK1 regulation and inhibition remains poorly understood. Using an integrated spectroscopic and computational approach, we uncover conformational transitions that govern substrate entry, catalysis, and inhibitor binding. Phosphorylation of Ser225 triggers regulatory loop rearrangements and salt bridge reshuffling, priming SK1 for membrane engagement and catalytic activity. We identify a previously uncharacterized catalytic intermediate featuring a distinct conformation with a highly dynamic lipid-binding loop 1 (LBL-1), sensitive to potent inhibitors such as PF-543. This inhibitor locks SK1 in an inactive state by restricting LBL-1 dynamics and globally stabilizing a non-catalytic conformation. Notably, SK1 forms functionally distinct dimers stabilized by ligand or membrane interactions, revealing a dynamic, multilayered regulatory mechanism governed by structural flexibility. These findings define a novel inhibitory mechanism and offer a structural framework for developing next-generation SK1-targeted therapeutics.

The bioactive lipid sphingosine-1-phosphate (S1P) plays a key role in regulating mammalian cell growth, survival, and migration^1^. It is essential for lymphocyte trafficking, immune responses, vascular and embryonic development, and bone homeostasis^2–4^. S1P is produced intracellularly by sphingosine kinase (SK) isoform 1 and 2, then released extracellularly to carry out its (patho)physiological functions^5^. Upon release, it activates its receptors (S1PR1–5), influencing numerous cellular processes^6^. Aberrant accumulation of S1P is linked to cancer progression and other diseases, including atherosclerosis, diabetes, and inflammatory disorders^2,4,5,7–11^. Hypoxia-induced upregulation of SK1 in endothelial and metastatic cells enhances S1P generation, promoting cell survival and migration^12^. ERK1/2 and PKC activation under hypoxic conditions, along with SK1 phosphorylation, induces conformational changes that facilitate membrane recruitment^13,14^. While a few SK1 crystal structures were solved over a decade ago^15–17^, key regulatory regions like the C-terminal tail and in some cases the loop bearing the phosphorylation site, Ser225, are missing or dissociated from the core protein. These regions are proposed to regulate conformational dynamics and the membrane recruitment^4,14^. Although existing structures provide valuable insights into the catalytic function and regulation of this important enzyme^4^, experimental validation using the full-length protein is needed to connect structural insights with functional dynamics. Thus, a comprehensive understanding of SK1’s structural and functional dynamics underlying its regulation and inhibition is crucial for guiding therapeutic strategies targeting S1P signaling in disease.

It is well established that plasma membrane translocation and enzymatic activity of SK1 are enhanced by ERK1/2-mediated phosphorylation of Ser225. However, the structural mechanism by which this modification alters SK1 conformation remains unresolved^4,14^. Ser225 resides on the regulatory loop (R-loop), which is positioned opposite the C-terminal domain (CTD) β-sandwich core from the lipid-binding site (Figs. 1d and 2i). A longstanding question is how phosphorylation of this loop enhances membrane recruitment—required for sphingosine (Sph) phosphorylation to S1P—and catalytic activity. Notably, Ser225 is solvent-exposed in all available structures^15,16^. The R-loop tip packs against the N-terminal domain (NTD), stabilized by Asp235, which inserts into a pocket formed by basic residues (His156, Arg162, His355; Fig. 2i). It is postulated that phosphorylation of Ser225 displaces the R-loop from its NTD interaction, thereby increasing protein flexibility^4^. The functionally important 20 C-terminal residues of SK1 are missing from current crystal structures^15,16^. Truncation beyond residue 363 renders SK1 constitutively active and unresponsive to phorbol 12-myristate 13-acetate (PMA)^18^, while also enhancing membrane localization independently of Ser225 phosphorylation.

The lipid-binding loop 1 (LBL-1; Fig. 1a, purple segment) plays a key role in membrane interaction^4,19^. A hydrophobic patch containing Leu194, Phe197, and Leu198 (Fig. 1d) mediates curvature-sensitive membrane binding, which is important in endocytosis^4,20,21^. Together with Lys27, Lys29, and Arg186, these residues serve as key determinants for specific and nonspecific interactions with anionic phospholipid-enriched membranes^19^. The mechanism of sphingosine entry into the substrate-binding pocket remains unclear^4^. However, structural data suggest that conformational changes involving helices 7 and 8 (LBL-1) regulate substrate access, forming a flap-like structure over sphingosine^15^. Moreover, LBL-1 binds to the calcium- and integrin-binding protein (CIB1) via Phe197 and Leu198, which is thought to mediate SK1 membrane translocation^4,22^.

Some studies suggest that SK1 functions as a dimer^4,11,19,23^, but structural insights into how dimerization affects catalytic efficiency and regulation are limited. A putative dimeric assembly has been proposed based on existing structures^4,14,15^, with the N-terminal domains possibly forming the dimer interface. Nonetheless, the conformational ensemble and dynamics of SK1 dimers remain poorly characterized.

As a dynamic enzyme, understanding SK1’s conformational landscape is vital for the rational design of allosteric modulators^24,25^. Detailed structural knowledge of how SK1 inhibitors, such as SKI-II and PF-543, engage the enzyme is still emerging^15–17^. PF-543 is a potent and selective SK1 inhibitor that acts via ATP-noncompetitive, substrate-competitive inhibition by mimicking sphingosine and occupying its binding pocket^16,26^. This prevents sphingosine access and phosphorylation. While PF-543 has proven invaluable in dissecting SK1 function, questions remain regarding its allosteric effects and ability to stabilize inactive conformations. Specifically, does PF-543 induce allosteric conformational changes beyond competitive blockade? How does it influence membrane-binding dynamics and the ATP-binding site, given that most structural studies exclude ATP?

Despite considerable progress, several key mechanistic and structural questions remain. These include the conformational transitions involved in allosteric regulation and membrane association, as well as the functional implications of dimerization. The effect of dimerization on activity, substrate affinity, or allosteric modulation remains speculative. Moreover, SK1’s dynamic changes upon ligand binding are still insufficiently defined, posing a challenge to structure-based drug design. Although crystal structures of SK1 exist in both inhibitor- and substrate-bound states, high-resolution structures with both sphingosine and Mg^2+^ATP are lacking, and conformational shifts during catalysis remain uncharacterized. To address these gaps, we employed double electron–electron resonance (DEER; also known as PELDOR) spectroscopy^27–31^, AlphaFold modeling^32^, and all-atom molecular dynamics (MD) simulations^33–37^ to uncover new SK1 conformational states and elucidate the mechanistic basis of its regulation and inhibition—advancing our understanding of this important therapeutic target.

## Results

Mechanistic characterization of SK1 requires identifying its conformational states during substrate binding and catalysis, understanding their dynamic interconversion, and determining how post-translational modifications and inhibitors reshape this landscape to stabilize active or inactive states. To this end, DEER distance distributions were measured for SK1 in its apo form, bound to sphingosine with and without Mg^2+^ATP, bound to the sphingosine analog FTY720 (an immunomodulatory drug), and in complex with the inhibitors SKI-II and PF-543. Most measurements were conducted in the presence of 0.1% Tween 20, a nonionic detergent used in prior studies^15,19^. Additional DEER experiments for select pairs were performed in the presence of large unilamellar vesicles (LUVs) composed of a plasma membrane-mimetic composition.

### Functional integrity of SK1 mutants

**Extended Data Figure 1.**
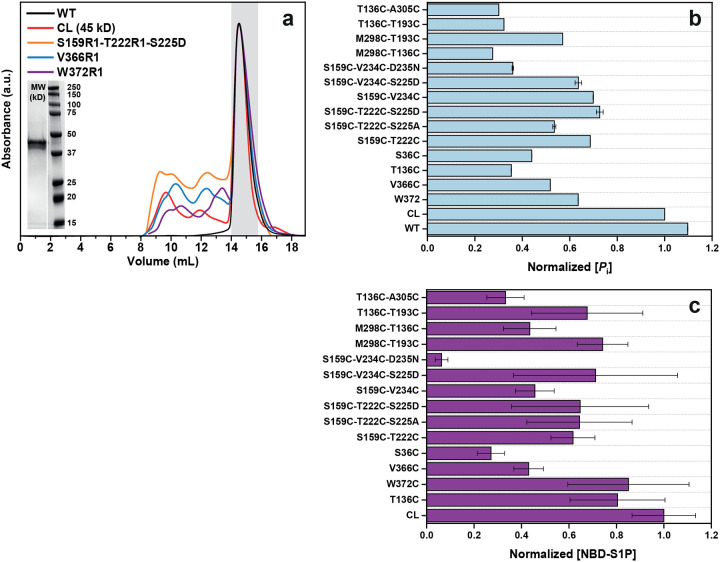
Functional integrity analyses. (**a**) Size exclusion chromatography profiles of the wild-type (WT), cys teine-less (CL), and representative spin-labeled mutants of SK1. (**b**) ATPase activity assay of the WT, CL, and spin-labeled DEER mutants, normalized to the CL SK1. (**c**) Fluorescence-based sphingosine kinase assay of the spin-labeled DEER mutants, normalized to the CL SK1.

To enable DEER spectroscopy, cysteine mutations were introduced into a cysteine-less (CL) SK1 background. The functional integrity of spin-labeled mutants was evaluated using two assays: a fluorescence-based sphingosine kinase assay with 15-NBD-sphingosine^38^ and an ATPase activity assay^39^ (Extended Data Fig. 1). Most DEER mutants retained activity comparable to the CL SK1. Notably, only the mutant containing the D235N substitution—which disrupts R-loop tethering—exhibited impaired sphingosine kinase activity (Extended Data Fig. 1c). In contrast, phosphomimetic (S225D) and phospho-null (S225A) mutations at Ser225 in the R-loop had no significant effect on enzyme activity.

### Dynamic lipid-binding loops regulate sphingosine entry, with PF-543 locking a closed conformation

**Extended Data Figure 2.**
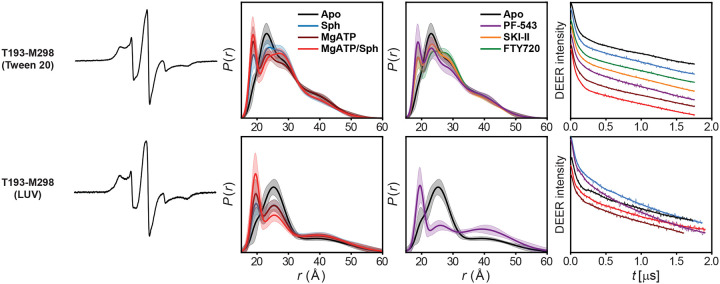
DEER data analysis for distance pairs probing the dynamics of the lipid-binding loops for sphingosine entry and binding. For each mutant, from left to right, CW EPR, distance distributions with confidence bands (2σ) about the best fit lines, and the primary DEER traces along with the fits are shown.

**Figure 1.**
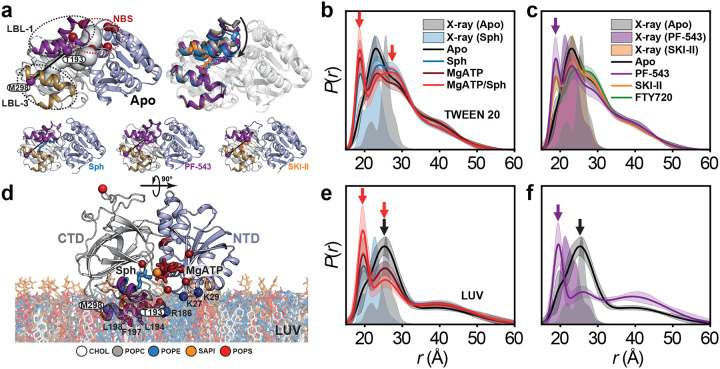
Dynamics of sphingosine entry, S1P release, and inhibition. (**a**) Structural comparison of SK1 lipid-binding loops in the apo (Protein Data Bank [PDB] code 3VZB-C), sphingosine-bound (3VZB-A), PF-543-bound (4V24-A), and SKI-II-bound (3VZC-D) states, with the NTD, CTD, LBL-1, and LBL-3 colored light blue, white, purple, and sand, respectively. Spin label pair for DEER dis tance measurement (LBL gating) and MgATP-binding residues (NBS) are represented by purple and dark red spheres, respectively. Tunnels (white) calculated using MOLEonline 2.5 in the apo structure suggest that subs trate entry from the membrane occurs through the LBL-1/LBL-3 gate, accessing the NBS occupied by MgATP. (**b**,**c**) Dis tance dis tributions *P*(*r*) in the presence of detergent (Tween 20). Confidence bands (2σ) are shown about the best fit lines. This band, which depicts the es timated uncertainty in *P*(*r*), reflects error associated with the fitting of the primary DEER trace. Predicted dis tributions from crystal structures of the apo, sphingosine-, PF-543-, and SKI-II-bound states are shaded in light gray, blue, purple, and orange, respectively. (**d**) Cartoon representation of MgATP/Sph-bound SK1 from our MD simulations, highlighting membrane-binding hydrophobic and electrostatic patches as purple sticks and dark blue spheres, respectively. (**e**,**f**) Dis tance dis tributions with liposomes (LUV). Red, purple, and black arrows indicate structural intermediates preferentially populated in the catalytic complex (MgATP/Sph), PF-543-bound, and apo states, respectively.

The mechanism by which sphingosine accesses its binding site in SK1 has remained unclear. Crystal structures (PDB code 3VZB) show sphingosine enclosed within the CTD, implying full lipid extraction (Fig. 1a,d). The only apparent entry is a narrow path through the ATP-binding site, which would require tail-first tunneling past polar residues. A more plausible mechanism involves dynamic opening of LBL-1, which form a flap over the binding site (Fig. 1d). Structural comparisons between apo and ligand-bound states suggest that LBL-1 rotates outward to expose inner hydrophobic surfaces and the β-sandwich core (Fig. 1a)^4,15^. However, crystal packing may restrict this movement, necessitating experimental validation. To investigate LBL dynamics during substrate and inhibitor binding, we monitored the T193–M298 distance (LBL-1 to LBL-3). In the apo state—especially with liposomes—measured distances matched the apo structure^15^, indicating an open LBL-1 (Fig. 1b,e and Extended Data Fig. 2). Upon sphingosine binding, a shift toward a shorter distance was observed, more prominently in liposomes, suggesting a closed conformation not captured in crystal structures. Binding of Mg^2+^ATP/sphingosine further stabilized this closed state (Fig. 1b,e), while Mg^2+^ATP alone partially shifted the equilibrium (Fig. 1e). These findings support a model where transient LBL-1 opening enables substrate recognition at the membrane interface, followed by substrate extraction and LBL-1 closure (Fig. 1d). In detergent, ligand binding increases LBL dynamics, especially in the catalytic Mg^2+^ATP/sphingosine complex, which samples both closed and open conformations (Fig. 1b, red arrows). This flexibility likely facilitates phosphoryl transfer and S1P release. Sphingosine analogs FTY720 and SKI-II induce similar LBL behavior (Fig. 1c), whereas PF-543 strongly favors the closed conformation and reduces LBL mobility, suggesting an inhibition mechanism that locks LBLs in a closed conformation and blocks substrate access (Fig. 1c,f). Thus, LBLs dynamics regulate sphingosine entry and S1P release, while potent inhibitors like PF-543 trap SK1 in a closed conformation, blocking substrate access.

### Ser225 phosphorylation reconfigures the R-loop into a catalytic state, modulated by PF-543

**Extended Data Figure 3.**
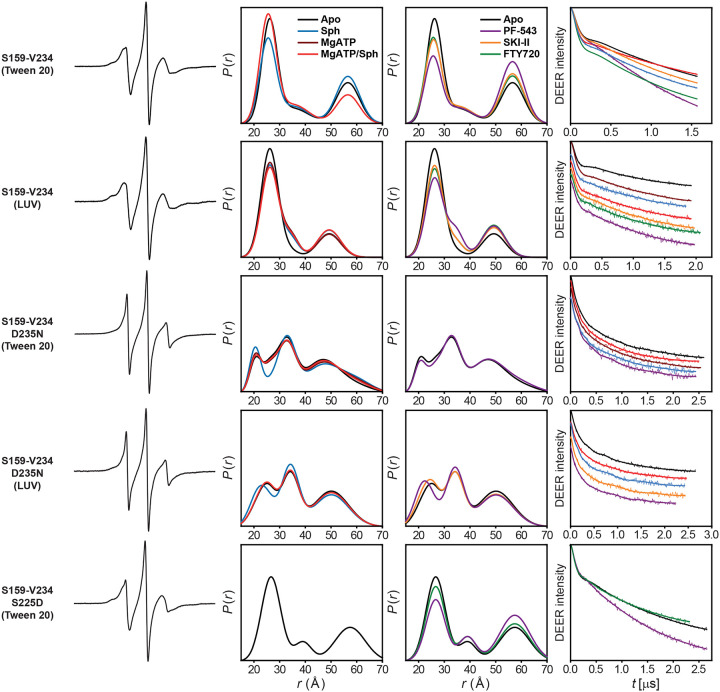
DEER data analysis for distance pair S159-V234, probing the dynamics of the regulatory loop. For each mutant, from left to right, CW EPR, distance distributions, and the primary DEER traces along with the fits are shown.

**Extended Data Figure 4.**
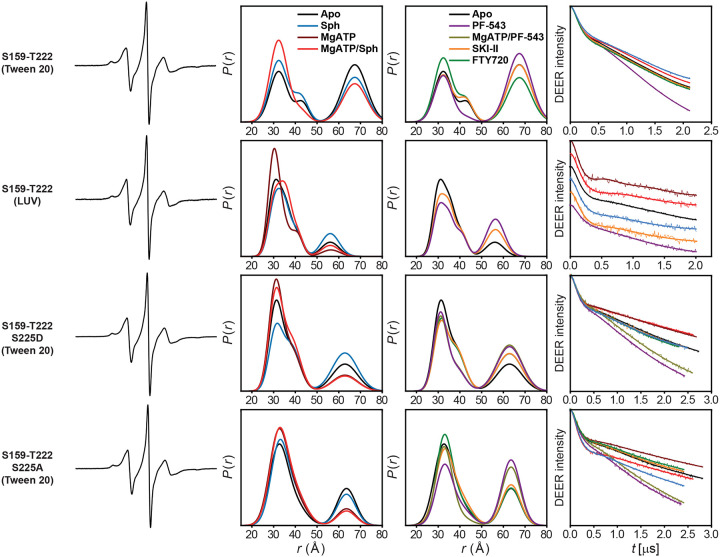
DEER data analysis for distance pair S159-T222, probing the dynamics of the regulatory loop. For each mutant, from left to right, CW EPR, distance distributions, and the primary DEER traces along with the fits are shown.

**Figure 2.**
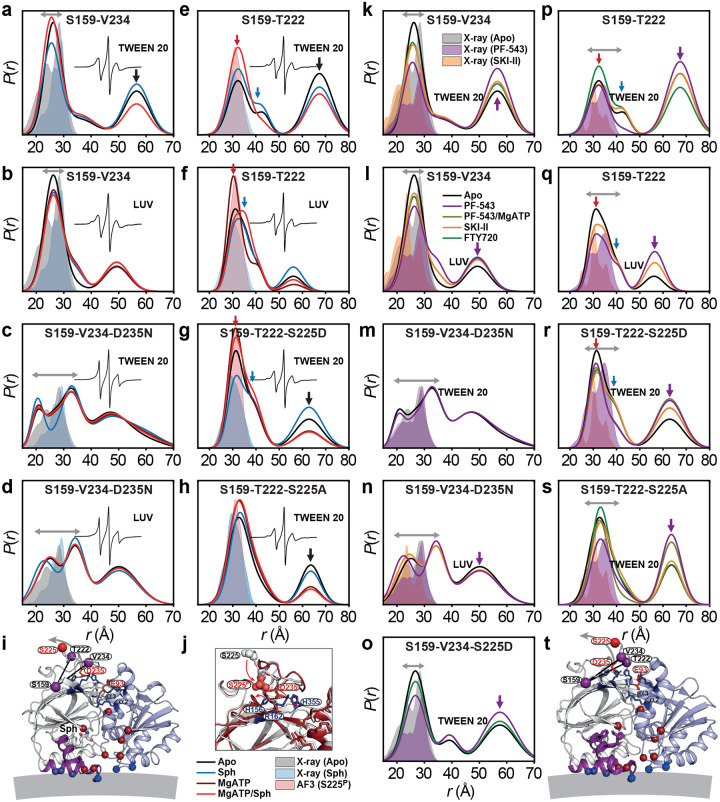
Ser225 phosphorylation reconfigures the R-loop into a catalytically competent s tate, modulated by PF-543. (**a**,**b**) Distance distributions *P*(*r*) probing R-loop dynamics with intact Asp235 forming a stabilizing salt bridge, (**c**,**d**) with the D235N mutation, which disrupts the salt bridge and enhances R-loop dynamics, supported by CW EPR spectra (insets). Double-headed gray arrows indicate the dynamic range of major conformational states. (**e**,**f**) Distance distributions probing dynamics near the Ser225 phosphorylation site with Ser225 mostly phosphorylated, (**g**) with phosphomimetic mutant S225D, or (**h**) phospho-null mutant S225A. Red and blue arrows indicate dominant alternative states at equilibrium, while black arrows denote populations likely arising from the dimeric state. Predicted distributions from the sphingosine-bound crystal structure and AlphaFold model of SK1 with Ser225 phosphorylation are shaded in light blue and red, respectively. (**i**) DEER distance pairs (purple spheres) mapped onto the sphingosine-bound structure, with NTD, CTD, and LBL-1 in light blue, white, and purple, respectively. Salt-bridge networks around the R-loop are shown as sticks. MgATP-binding and membrane-interacting residues are shown as dark red and blue spheres. (**j**) Local R-loop rearrangement and salt-bridge reshuffling upon Ser225 phosphorylation (AlphaFold 3 [AF3] model). (**k–s**) Distance distributions probing R-loop dynamics in the presence of inhibitors and the sphingosine analog FTY720. (**t**) Distance pairs mapped onto the PF-543-bound crystal structure.

Phosphorylation at Ser225, a key regulator of SK1’s activity and membrane localization, induces conformational changes that remain incompletely characterized. Ser225 lies exposed within the R-loop, on the reverse side of the CTD β-sandwich, away from the lipid-binding site (Fig. 2i). The R-loop tip contacts NTD helices α3/α4 and is stabilized by Asp235, which inserts into a β-sandwich pocket formed by His156, Arg162, and His355 (Fig. 2i). It is proposed that phosphorylation displaces Asp235, altering R-loop–NTD interactions and aligning membrane-facing residues in the NTD and CTD (Fig. 2i, blue spheres) for optimal membrane binding^4,14^. To monitor the R-loop dynamics, the S159-V234 distance pair reports on R-loop movement near Asp235 (Fig. 2 and Extended Data Fig. 3). Across WT conditions, with minor variations, the dominant distance population corresponds to those observed in the apo and sphingosine-bound structures, indicating a stable Asp235 salt bridge critical for catalysis (Fig. 2a,b)^15^. Interestingly, the D235N mutation enhances R-loop dynamics and shifts the population toward longer distances, indicating salt bridge disruption (Fig. 2c,d). This effect is also evident in CW-EPR spectra compared with the WT protein^40^. The S159–T222 pair monitors changes near Ser225 (Fig. 2 and Extended Data Fig. 4), revealing three distinct conformations: one matching the sphingosine-bound crystal structure (red arrow), and two representing novel intermediates (blue and black arrows; Fig. 2e,f). Interestingly, AlphaFold modeling of phosphorylated SK1 (Ser225^P^) predicts a conformation where Ser225^P^ forms a salt bridge with His156/Arg162, replacing Asp235, while the Asp235–His355 salt bridge remains intact (Fig. 2j). Importantly, this configuration matches the experimentally determined DEER ensemble in the Mg^2+^-ATP-bound state (Fig. 2f). Mass spectrometry confirms Ser225 phosphorylation in our constructs, consistent with the DEER data. As expected, the phosphomimetic S225D shifts the equilibrium toward the phosphorylated conformation (Fig. 2g, red arrow), whereas the phospho-null S225A mutation stabilizes an intermediate conformation (between the red and blue arrows) distinct from WT, S225D, and phosphorylated AlphaFold model (Fig. 2h).

**Extended Data Figure 5.**
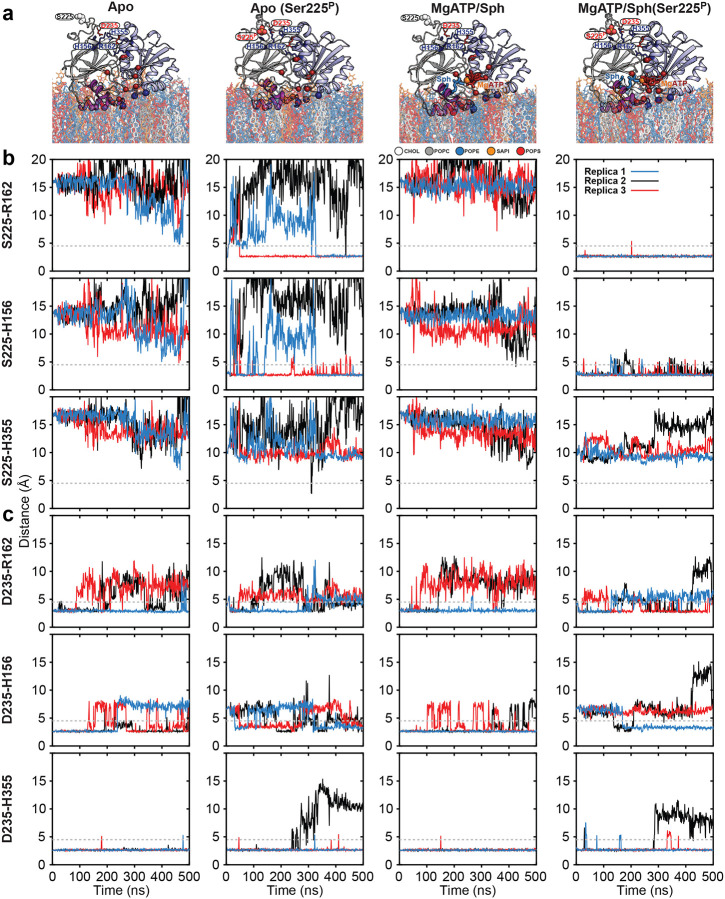
Molecular dynamics simulations of SK1 bound to plasma membrane (PM) lipid bilayers. (**a**) Four MD simulation sys tems, each performed in three independent replicates. (**b**,**c**) Time series of the dis tances between three basic residue side chains and either Ser225/Ser225P or Asp235 during simulations, used to monitor salt bridge formation between the R-loop and the strand pair connecting the NTD to the CTD. Phosphorylation of Ser225 stabilizes salt bridges to His156 and Arg162, reconfiguring the R-loop. Binding of subs trates (MgATP and Sph) mutually and allos terically stabilizes this R-loop conformation. Regardless of the condition, Asp235 consis tently maintains a tight interaction with His355, while the salt bridge with His156 is less stable and becomes disrupted upon Ser225 phosphorylation.

To further investigate the rearrangement of salt bridges between the R-loop and the strand pair connecting the NTD to the CTD upon Ser225 phosphorylation, we conducted all-atom equilibrium MD simulations in the presence and absence of ligands and R-loop phosphorylation (Extended Data Fig. 5). MD simulations further show that Ser225 phosphorylation stabilizes salt bridges with His156/Arg162 and reconfigures the R-loop, especially in the presence of substrates (Extended Data Fig. 5b and Supplementary Video 1). Thus, consistent with the DEER measurements (Fig. 2e–g), substrate binding mutually and allosterically stabilizes this R-loop conformation. Although phosphorylation alone can form these bridges, they are less stable without substrates (Extended Data Fig. 5b and Supplementary Video 2). Asp235 consistently maintains interaction with His355, but its salt bridge with His156 weakens upon phosphorylation (Extended Data Fig. 5c). These results underscore Ser225 phosphorylation as a key allosteric switch for adopting a catalytically competent conformation.

A third, long-distance R-loop state is frequently observed in detergent (black arrows, Fig. 2a,e,g,h and Extended Data Figs. 3,4), potentially representing an oligomeric form of SK1 (see Discussion). Interestingly, FTY720 and inhibitors modulate R-loop conformations (Fig. 2k–s). PF-543 prominently shifts the R-loop toward this long-distance state (purple arrows), even with Mg^2+^ATP present, suggesting this conformation is part of its inhibitory mechanism. This effect is abolished by the D235N mutation (Fig. 2m,n), confirming Asp235’s central role in SK1 catalytic and inhibition mechanisms. MD simulations support a stable Asp235–His355 interaction across conditions (Extended Data Fig. 5c). Additional residues, such as Asn89 and Glu93 (Fig. 2i)^4^, may also contribute to phosphorylation-induced membrane targeting. Asn89, in particular, forms potentially stabilizing cross-domain hydrogen bonds that may help align the NTD and CTD for optimal membrane binding.

### SK1 adopts a novel catalytic conformation inhibited by PF-543

**Extended Data Figure 6.**
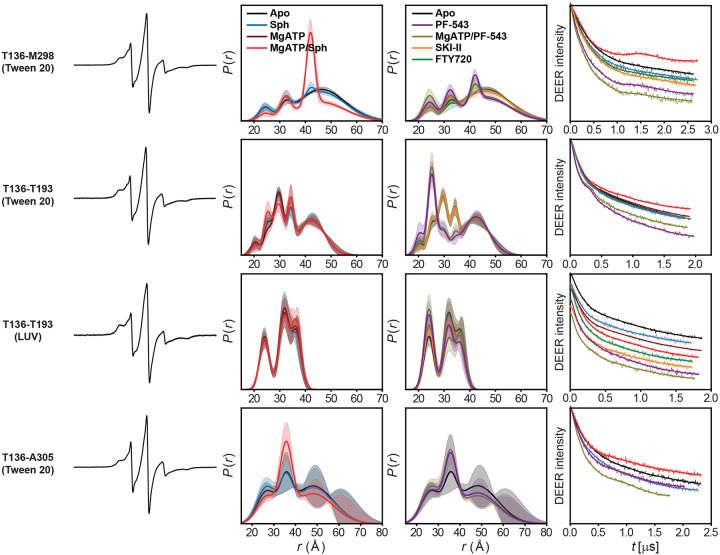
DEER data analysis of dis tance pairs probing the relative orientation and dynamics of the N-terminal and C-terminal domains, regulated by subs trate and inhibitor binding, as well as S1P synthesis. For each mutant, from left to right, CW EPR, distance distributions with confidence bands (2σ) about the best fit lines, and the primary DEER traces along with the fits are shown.

**Figure 3.**
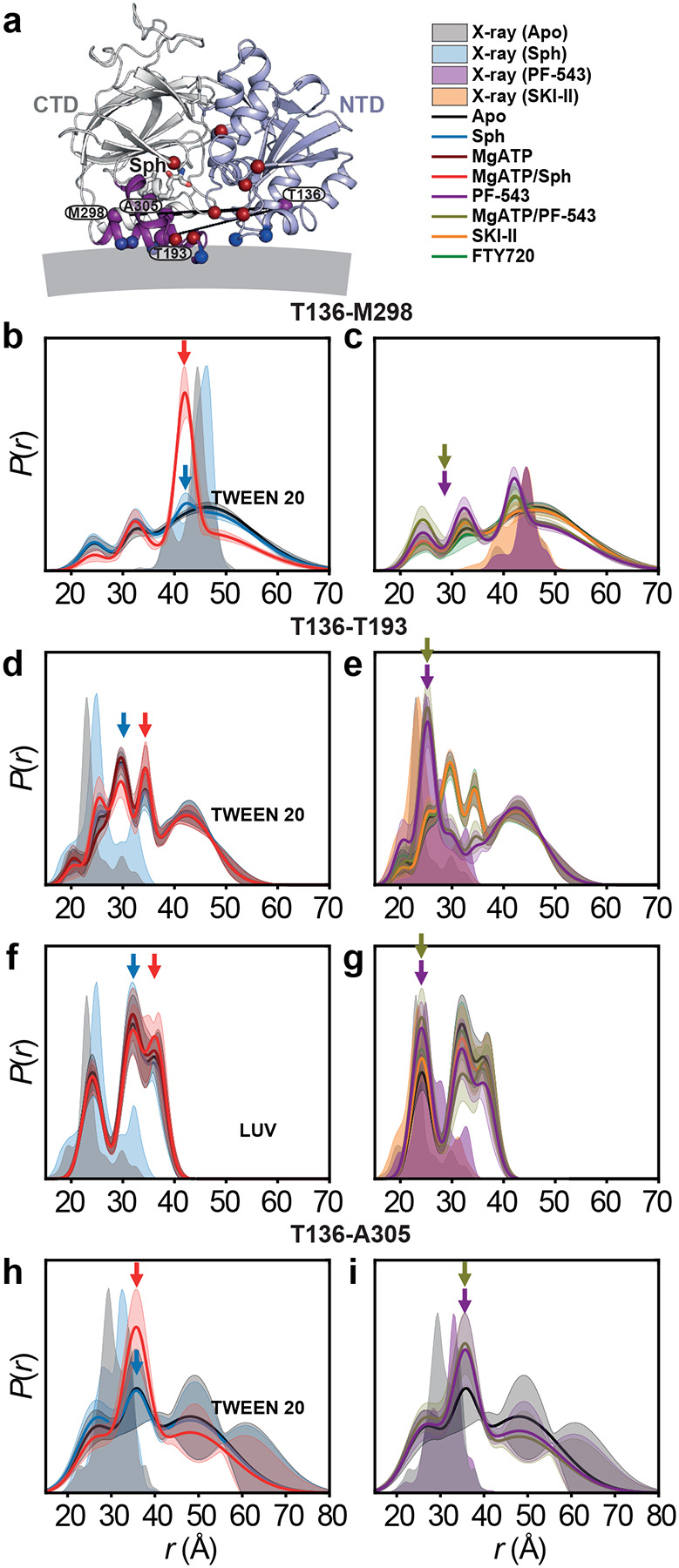
SK1 adopts a novel catalytic conformation with a dynamic LBL-1, inhibited by PF-543. (**a**) DEER dis tance pairs (purple spheres) probing the catalytic core mapped onto the sphingosine-bound structure, with the NTD, CTD, and LBL-1 shown in light blue, white, and purple, respectively. MgATP-binding and membrane-interacting residues are depicted as dark red and blue spheres. (**b–i**) Distance dis tributions *P*(*r*) with ligands and inhibitors. Red, blue, purple, and dark yellow arrows indicate structural intermediates predominantly populated in the catalytic complex (MgATP/Sph), Sph-bound, PF-543-bound, and MgATP/PF-543 states, respectively.

We introduced interdomain distance pairs between the NTD and CTD to investigate their relative orientation and dynamics, which are regulated by ligand and inhibitor binding, as well as S1P synthesis (Fig. 3 and Extended Data Fig. 6). Sphingosine, its analogs, and inhibitors bind to the CTD, while Mg^2+^ATP binds to the NTD at the domain interface (Fig. 3a). Except for the T136-T193 pair, the catalytic complex with Mg^2+^ATP/sphingosine is well-structured (Fig. 3b,h). The protein remains highly dynamic in the apo and sphingosine-bound states. The catalytic complex, which has never been structurally studied before, adopts a conformation distinct from the apo and sphingosine-bound crystal structures (Fig. 3). Interestingly, the T136-T193 pair, which includes the catalytic LBL-1 region, remains highly dynamic in all states, including the catalytic complex (Fig. 3d,f), and deviates significantly from the apo and sphingosine-bound crystal structures. Additionally, for two interdomain distance pairs, with one site located on the LBLs in the CTD (T136-T193 and T136-M298), three intermediates emerge. The longest-distance intermediate is more populated in the catalytic complex (Fig. 3b,d), while PF-543-bound state preferentially populates shorter-distance intermediates, with or without Mg^2+^ATP (Fig. 3c,e,g). For the T136-T193 pair, this domain configuration aligns with the PF-543-bound crystal structure, suggesting that PF-543 stabilizes a non-catalytic conformation, unlike sphingosine or SKI-II, regardless of Mg^2+^ATP binding. In contrast, the catalytic complex maintains LBL-1 flexibility (Figs. 1b and 3d), which likely supports S1P production and release. These findings provide additional structural insights into the inhibition mechanism of PF-543.

### Ser225 phosphorylation may release the C-terminal tail

**Extended Data Figure 7.**
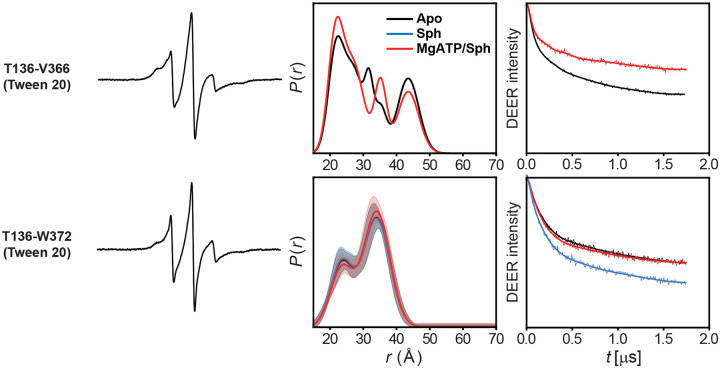
DEER data analysis for distance pairs probing the dynamics of the C-terminal tail. For each mutant, from left to right, CW EPR, distance distributions with confidence bands (2σ) about the best fit lines, and the primary DEER traces along with the fits are shown.

**Figure 4.**
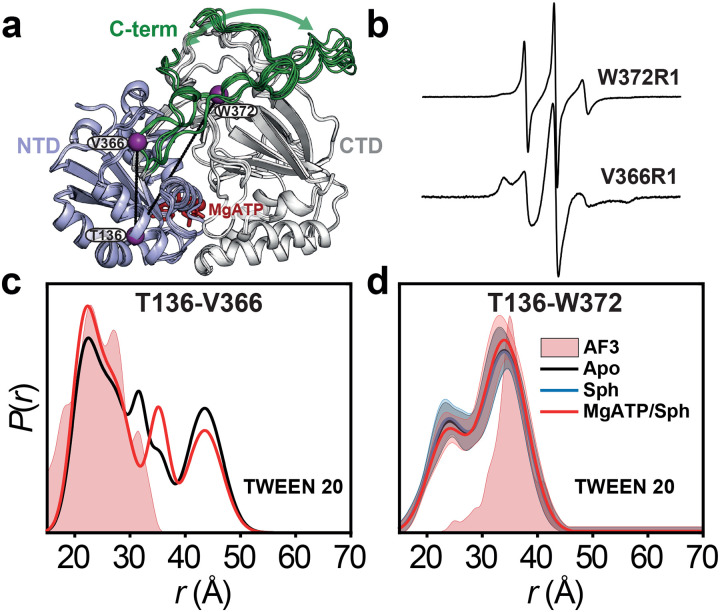
Ser225 phosphorylation may release the C-terminal tail. (**a**) An ensemble of full-length AlphaFold 3 models bound to MgATP with an intact C-terminal tail (green) reveals a highly dynamic tail. DEER dis tance pairs (purple spheres) probing the tail are mapped onto one model. (**b**) CW-EPR spectra of two singly labeled positions on the loop indicate that the N-terminus is in contact, while the C-terminus is untethered and highly dynamic. (**c**,**d**) Distance dis tributions *P*(*r*) probing the dynamics of the tail. Predicted dis tributions from the AlphaFold 3 model of SK1 are shaded in light red.

The C-terminal tail (residues 365–384), absent from all crystal structures, harbors known protein interaction sites and plays a key regulatory role (Fig. 4a)^4,14,18^. Interestingly, its interaction with the long, twisted strand pair that connects the NTD and CTD—implicated in R-loop anchoring—has been postulated (see Discussion)^14^. In this context, Ser225 phosphorylation-induced structural transitions in the R-loop may promote C-terminal tail release, facilitating membrane targeting. CW EPR spectrum of spin-labeled Val366 at the tail’s N-terminus showed intermediate spin label motion^40^, indicating contact with the core protein (Fig. 4b)^19^. In contrast, the Trp372R1 spectrum exhibited sharper, narrower lines, suggesting a more flexible conformation and greater mobility for this site and subsequent residues. Given predominant Ser225 phosphorylation in our constructs, this supports a model in which the tail is released from its potential tethering to the core protein. DEER distance distributions between these residues and Thr136 suggest that the C-terminal tail adopts multiple conformations (Fig. 4c,d and Extended Data Fig. 7), with functional implications that warrant further investigation.

### SK1 forms dimers via distinct interfaces modulated by ligands and membrane context

**Extended Data Figure 8.**
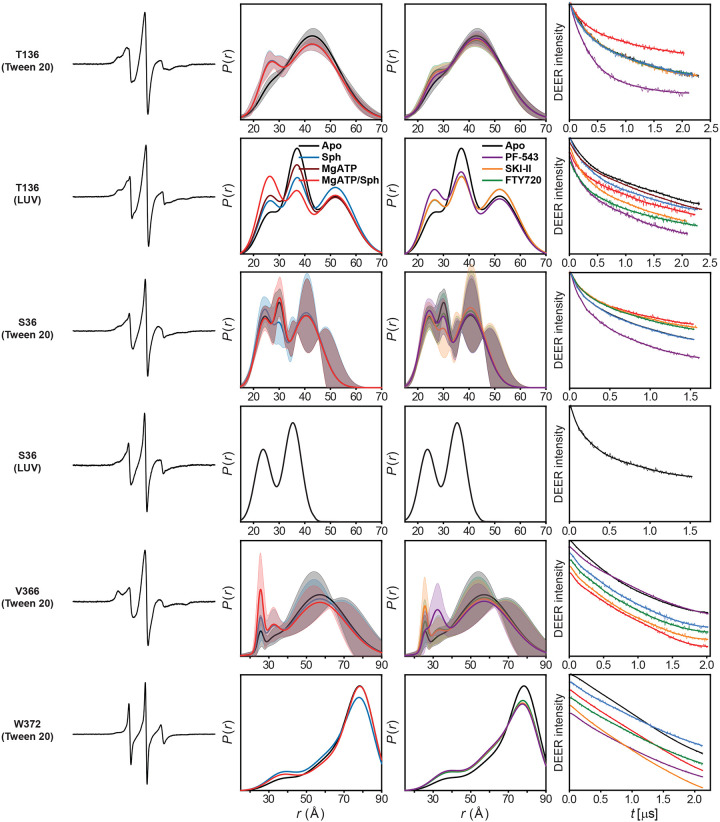
DEER data analysis of singly labeled SK1 protomers probing its oligomeric state, quaternary structure, and dynamics. For each mutant, from left to right, CW EPR, distance distributions with confidence bands (2σ) about the best fit lines, and the primary DEER traces along with the fits are shown.

**Figure 5.**
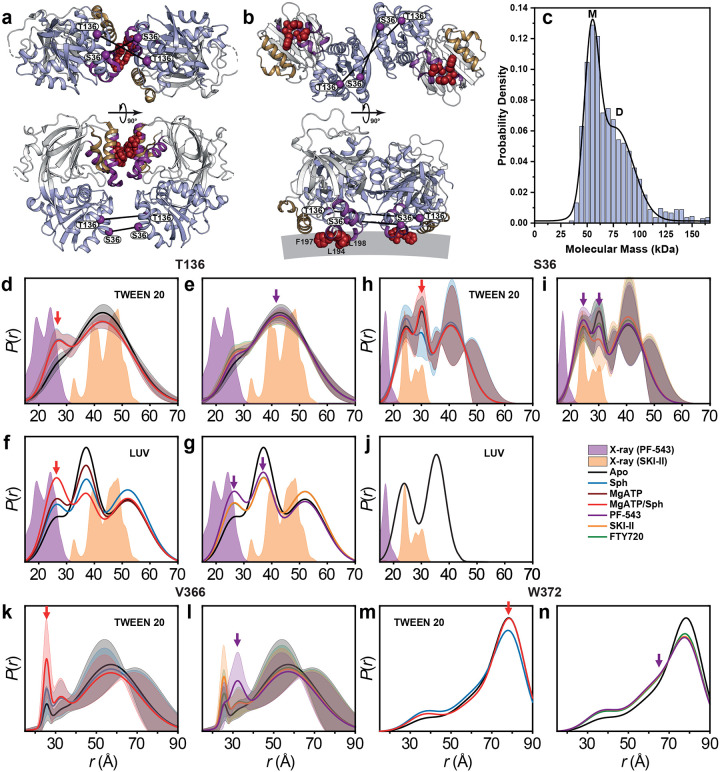
SK1 forms dimers through dis tinct interfaces influenced by ligands and membrane context. (**a**) Cartoon representation of the dimeric PF-543–bound crystal structure (PDB code 4V24) and (**b**) the dimeric SKI-II–bound crystal structure (PDB code 3VZC, chains B and C), with the NTD, CTD, LBL-1, and LBL-3 shown in light blue, white, purple, and sand, respectively. Membrane-binding hydrophobic patches are shown as dark red spheres, and spin-labeled positions as purple spheres. (**c**) Mass photometry analysis of WT SK1 (100 nM) in detergent-free buffer reveals a mixture of monomeric (M) and dimeric (D) forms. (**d–n**) Dis tance dis tributions *P*(*r*) of singly labeled SK1 in the presence of ligands and inhibitors. PF-543 shifts the equilibrium away from the catalytic complex (MgATP/Sph), stabilizing distinct dimeric conformations. Red and purple arrows indicate dimeric states populated in the catalytic complex and PF-543–bound conditions, respectively.

SK1 dimerization has been demonstrated in cells using immunoprecipitation assays^23^. Structural data also reveal two distinct potential dimerization interfaces—mediated by either the CTD or NTD domains—in SK1 bound to inhibitors PF-543 (PDB: 4V24; Fig. 5a)^16^ and SKI-II (PDB: 3VZC; Fig. 5b)^4,14,15^, respectively. However, these putative dimer interfaces have yet to be confirmed by biophysical methods and require further investigation. Using mass photometry, we observed SK1 dimerization (Fig. 5c). Notably, in the PF-543-bound structure (Fig. 5a), the hydrophobic residues involved in membrane binding (Leu194, Phe197, Leu198) mediate dimerization, whereas in the SKI-II-bound structure (Fig. 5b), these same residues remain exposed and aligned for membrane interaction. To probe the quaternary structure of the SK1 dimer in both detergent and liposome-bound states, we used DEER spectroscopy with singly labeled SK1 protomers (Fig. 5d–n). The SK1 dimer adopts a more structured conformation when bound to unilamellar vesicles compared to detergent (Fig. 5d–g). Distance populations consistent with both CTD- and NTD-mediated dimer models were observed at labeled sites T136 and S36 under different ligand-bound conditions. For S36, the predicted distance in the CTD-mediated dimer is too short to be captured in the DEER distance ensemble (Fig. 5h,i), while the NTD-mediated state appears under multiple conditions. Interestingly, for position T136, the CTD-mediated dimer is more populated in the catalytic complex with Mg^2+^ATP/sphingosine (Fig. 5f). Importantly, primary DEER traces showed increased modulation depth under the PF-543-bound condition (Extended Data Fig. 8), indicating enhanced oligomerization under this condition. At position V366 on the C-terminal tail, DEER revealed a well-defined short-distance population in the catalytic complex, alongside a broad, long-distance component. Due to the absence of resolved structural data for the C-terminal tail, the structural significance remains unclear. However, if these intermediates are functionally relevant, they may explain the CW EPR spectrum at this site (Fig. 4b), which suggests a partially tethered or in contact conformation of the tail’s N-terminal region rather than a fully untethered state observed for the position W372. Intriguingly, PF-543 (purple arrow) and the catalytic complex (red arrow) exert opposing effects on tail dynamics (Fig. 5k–n), further supporting PF-543’s role in stabilizing a non-catalytic conformation. Nonetheless, the functional consequences of SK1 dimerization remain to be elucidated.

## Discussion

**Extended Data Figure 9.**
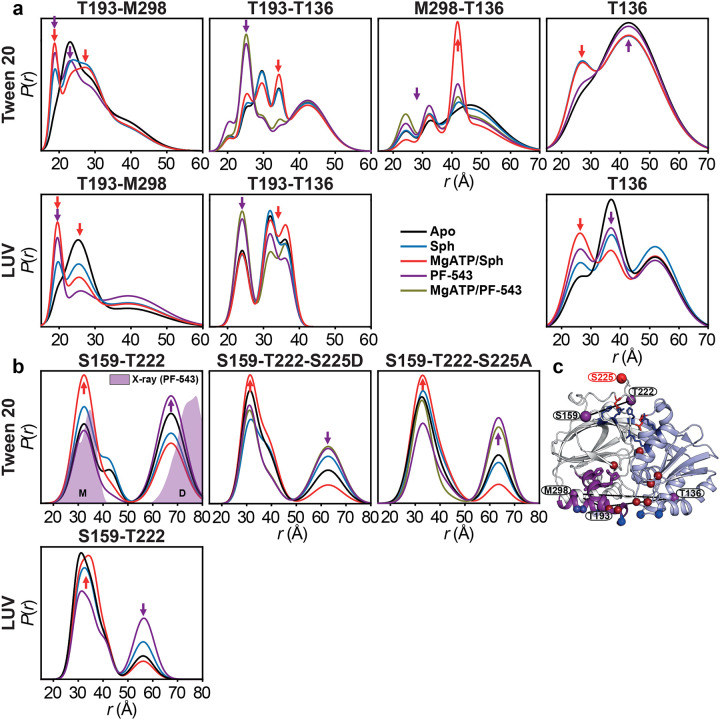
The catalytically active SK1 adopts a novel conformation featuring a highly dynamic LBL-1 region that is sensitive to PF-543, which globally stabilizes a non-catalytic state. (**a**) Distance distributions *P*(*r*) for SK1 pairs probing the catalytic core in the presence of detergent (Tween 20) or liposomes. Red and purple arrows highlight intermediates predominantly populated in the catalytic complex and PF-543-bound states, respectively. (**b**) Distance distributions for the SK1 pair S159-T222 probing the regulatory loop, along with phosphomimetic (S225D) and phospho-null (S225A) mutants. The distribution predicted from the PF-543-bound dimeric crystal structure (PDB code 4V24) is shaded in purple. Monomeric (M) and dimeric (D) contributions are indicated. (**c**) DEER dis tance pairs (purple spheres) mapped onto the PF-543-bound crystal structure, with the NTD, CTD, and LBL-1 shown in light blue, white, and purple. MgATP-binding and membrane-interacting residues are represented by dark red and blue spheres, respectively.

**Extended Data Figure 10.**
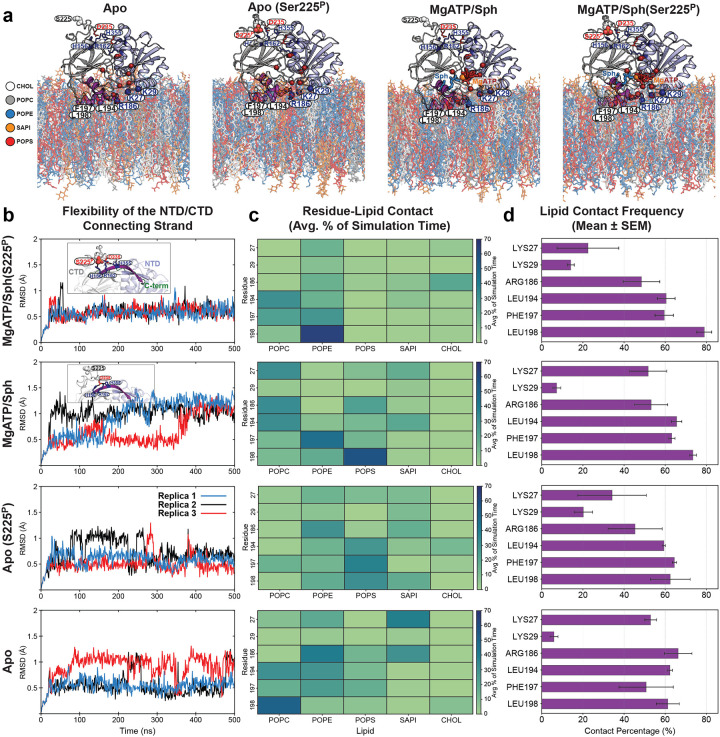
MD analysis of the flexibility of the NTD/CTD connecting s trand pair and membrane interactions of key membrane-interfacing determinants. (**a**) Four MD simulation conditions, each performed in three independent replicates. Membrane-interacting residues are shown as dark blue (basic) and purple (hydrophobic) spheres. (**b**) Flexibility of the NTD/CTD connecting strand (residues 350–362, shown in purple in the inset) in ligand-bound SK1 is significantly reduced upon Ser225 phosphorylation, likely due to the formation of stable salt bridges (i.e., Ser225P:Arg162, Ser225P:His156, and Asp235:His355; see Extended Data Fig. 5). (**c**) Lipid interaction specificity of membrane-interfacing residues, plotted as the average contact percentage of total simulation time, reveals dis tinct interaction patterns for phosphorylated versus non-phosphorylated SK1. Electros tatic interactions between basic residues and anionic lipids are significantly diminished in the MgATP/Sph-bound phosphorylated protein, while hydrophobic interactions increase. In the apo state, Arg186 and Lys27 preferentially interact with SAPI over POPS, with Arg186 also contacting POPE. In Apo(Ser225P) state, the hydrophobic patch shows increased contact with POPS. (**d**) Overall, the lipid contact frequency of the hydrophobic patch and Arg186 remains high across conditions, whereas Lys27 lipid interactions are significantly reduced upon Ser225 phosphorylation. Lys29 lipid interactions show a slight increase.

Using an integrated spectroscopic and computational approach, this study reveals new mechanistic insights into SK1, uncovering a dynamic, multilayered regulatory mechanism in which structural flexibility governs both catalysis and inhibition. A key finding is that lipid-binding loop (LBL) dynamics gate sphingosine entry into the active site, with the potent inhibitor PF-543 locking the LBLs in a closed, less dynamic conformation that blocks ligand exchange (Extended Data Fig. 9a). A major breakthrough is the discovery that phosphorylation of Ser225 may trigger a reconfiguration of the R-loop and the overall SK1 structure into a catalytically competent state. Comprehensive MD simulations reveal a reshuffling of salt bridges involving positively charged residues on the strand pairs connecting the two SK1 lobes—potentially facilitating membrane engagement (Extended Data Figs. 5 and 10) and release of the C-terminal tail. These simulations uncover phosphorylation- and ligand-dependent shifts in the balance between electrostatic and hydrophobic interactions in membrane engagement, suggesting a dynamic mechanism by which SK1 senses and responds to the membrane environment (Extended Data Fig. 10). The catalytically active SK1 adopts a previously uncharacterized conformation—distinct from known apo or ligand-bound structures—featuring a highly dynamic LBL-1 region that is sensitive to PF-543, which globally stabilizes a non-catalytic state (Extended Data Fig. 9). Furthermore, SK1 forms dimers through distinct ligand- and membrane-dependent interfaces.

Defining the molecular inhibitory mechanism of potent inhibitors like PF-543 is crucial for developing more effective therapeutic modulators. A key finding of this study is the identification of a previously uncharacterized catalytic complex. Probed through three distance pairs—T193–T136, T193–M298, and T136–M298 (Extended Data Fig. 9a)—this complex features a highly dynamic catalytic core involving LBL-1 and LBL-3, which wrap around and position the substrate within the CTD for phosphoryl transfer, alongside the domain interface encompassing the Mg^2+^ATP binding site. This dynamic core appears to transition through three intermediates essential for both phosphoryl transfer and release of synthesized S1P. Notably, PF-543—regardless of Mg^2+^ATP presence—shifts the equilibrium among these intermediates, effectively inhibiting catalysis or ligand exchange (Extended Data Fig. 9a). This inhibitory effect extends to the dimeric conformation of SK1 (Fig. 5 and Extended Data Fig. 9) and the regulatory loop (Extended Data Fig. 9b).

**Figure 6.**
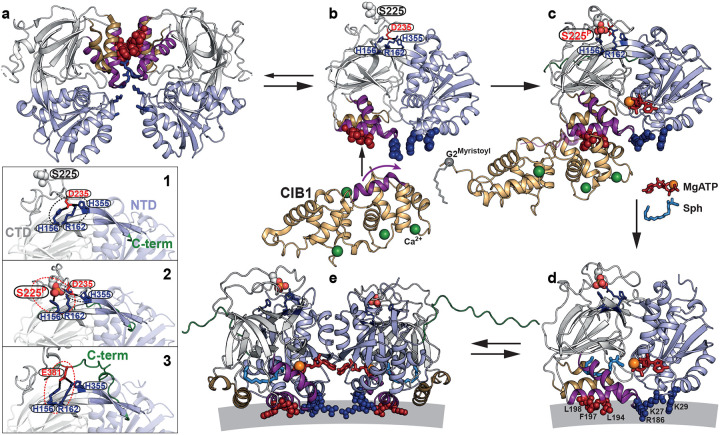
Proposed regulatory mechanism of SK1. (**a**) In its resting state, SK1 may form a CTD-mediated dimer resembling the PF-543-bound crystal structure (PDB code 4V24), representing a catalytically inactive conformation where key membrane-interacting residues (Leu194, Phe197, and Leu198 in LBL-1; red spheres) are buried within the dimer interface. (**b**) CIB1 (PDB code 1XO5) binds to monomeric SK1 (PDB code 3VZB-A) by recognizing the now-exposed hydrophobic residues in LBL-1. Upon calcium binding, CIB1 undergoes a structural transition that exposes a membrane-targeting N-terminal myris toyl group and forms a groove for SK1 interaction via unfolding of a C-terminal helix (**c**; AF3 model). Simultaneously, phosphorylation of Ser225 partially displaces Asp235 in the R-loop, reshaping the salt bridge network with positively charged residues in the strand pair linking the NTD and CTD (inset: transition from state **1** [PDB code 3VZB-A] to state **2** [AF3 model]). This rearrangement drives conformational changes that stabilize a catalytically active state for sphingosine extraction and Mg^2+^ATP binding (**d**; AF3 model). Conserved acidic residues in the C-terminal tail (e.g., Glu381) may also interact with the same positively charged residues in the strand pair (inset: state **3**; AF3 model), stabilizing an inactive conformation—potentially by misaligning membrane-interfacing elements, similar to the PF-543-bound state. In this context, Ser225 phosphorylation and R-loop rearrangement may facilitate release of the C-terminal tail, a conformation supported by our experimental observations. (**e**) Additionally, the membrane may promote formation of an NTD-mediated dimeric state, as observed in experimental data (AF3 model superimposed on PDB code 3VZC-B/C).

Interestingly, PF-543 appears to stabilize a dimeric configuration similar to that observed in the PF-543 crystal structure (Figs. 5a and 6a), suggesting a functionally relevant state rather than a crystallographic artifact. A long-distance intermediate, potentially corresponding to this dimeric form, becomes more populated upon PF-543 binding (purple arrows; Extended Data Fig. 9b), indicating a catalytically inactive SK1 state in which key membrane-interfacing elements (Leu194, Phe197, and Leu198 in LBL-1) are sequestered within the dimeric interface (Fig. 6a). The presence of a CTD-mediated dimeric structure provides a mechanistic rationale for the CIB1-dependent membrane translocation of SK1, as CIB1 engages SK1 through the same hydrophobic residues in LBL-1 (Fig. 6b). Upon calcium binding, CIB1 undergoes a structural transition that exposes both a membrane-targeting N-terminal myristoyl group and a groove for partner protein interaction—the latter formed by the unfolding of a C-terminal helix (Fig. 6c). Simultaneously, Ser225 phosphorylation partially replaces Asp235 in the R-loop, reconfiguring salt bridges with positively charged residues on the strand pair connecting the two domains (Fig. 6 inset, states 1 to 2; Extended Data Fig. 5). This reconfiguration may induce conformational changes in the strand pair that stabilize a catalytically competent state for sphingosine extraction from the membrane and Mg^2+^ATP binding at the catalytic core (Fig. 6d). Notably, our MD simulations indicate that with phosphorylation and Mg^2+^ATP/sphingosine binding, one of the connecting strands becomes significantly more stable, potentially stabilizing the catalytic complex (Extended Data Fig. 10b). This strand connects to the SK1 C-terminal tail. Consistently, in our DEER constructs where Ser225 is predominantly phosphorylated or mutated to the phosphomimetic S225D, this R-loop conformation appears particularly stabilized in the presence of MgATP or MgATP/Sph (Fig. 2e–g). Additionally, MD analyses reveal distinct membrane interaction patterns for key membrane-interfacing determinants in phosphorylated versus non-phosphorylated SK1 (Extended Data Figs. 10c and 10d). Electrostatic interactions between basic residues and anionic lipids are significantly diminished in the MgATP/Sph-bound phosphorylated protein, while hydrophobic interactions increase. In the apo state, Arg186 and Lys27 preferentially interact with SAPI over POPS, with Arg186 also contacting POPE. In the phosphorylated apo state, the hydrophobic patch shows increased contact with POPS. Overall, the lipid contact frequency of the hydrophobic patch and Arg186 remains high across different states, whereas lipid interactions involving Lys27 are significantly reduced, and those involving Lys29 are slightly increased upon Ser225 phosphorylation (Extended Data Fig. 10d). The residues involved in this long-range allosteric transmission will be further investigated through mutational analysis and conformational dynamics studies using DEER spectroscopy. Conserved acidic residues in the C-terminal tail (e.g., Glu381) have also been proposed to interact with the same positively charged residues in the connecting strand pair (Fig. 6 inset, state 3)^14^, thereby stabilizing an inactive conformation—potentially by misaligning key membrane-interacting elements, similar to the conformation stabilized by PF-543 (Fig. 3e,g). In this context, Ser225 phosphorylation and R-loop reconfiguration may release the C-terminal tail, a conformation we observe experimentally (Fig. 4), though this observation warrants further investigation. Additionally, the membrane itself may stabilize the NTD-mediated dimeric conformation observed experimentally (Figs. 5 and 6e).

## Online Methods

No statistical methods were used to predetermine sample size. The experiments were not randomized. The investigators were not blinded to allocation during experiments and outcome assessment.

### Site-directed mutagenesis

Codon-optimized human SK1 (GenScript) was cloned into a pFastBac-1 vector encoding an N-terminal 6×His tag (44.9 kDa). The seven cysteine residues in SK1 were mutated to alanine via site-directed mutagenesis using complementary oligonucleotide primers, yielding the CL variant. This construct served as the template for introducing single cysteine or double-cysteine pairs and background mutations. Substitution mutations were generated via single-step PCR, in which the entire plasmid was replicated from a single mutagenic primer. SK1 mutants were verified by sequencing with both pFastBac forward and reverse primers (Azenta) to confirm the desired mutations and absence of off-target changes. Mutants are designated by the native residue and sequence position, followed by the substituted residue.

### Expression, purification, and labeling of SK1

Wild-type (WT), cysteine-less (CL), and single- and double-cysteine SK1 mutants were expressed using standard Invitrogen protocols with the pFastBac system. *Spodoptera frugiperda* Sf9 cells (Gibco, ThermoFisher Scientific) were infected at a density of 2–2.5 × 10^6^ cells/mL and incubated for 66–72 hours at 27 °C in a shaking incubator using 2 L disposable flasks. Cells were harvested by centrifugation and stored at −80 °C. For lysis, cell pellets were resuspended in buffer (50 mM Tris·HCl, pH 7.8, 200 mM NaCl, 20 mM imidazole, and 10% [vol/vol] glycerol) at 2.6 mL per gram of cells. The buffer was supplemented with 0.5 mM TCEP, 0.9 mM (0.1% [vol/vol]) Tween 20 (Sigma), one EDTA-free protease inhibitor cocktail tablet (Roche), and 1 mM PMSF. The suspension was lysed by sonication, and cell debris was removed by centrifugation at 96,000 × g for 1 hour. The supernatant was incubated with 1.5 mL (bed volume) of His60 Ni-IDA Superflow resin (Takara) at 4 °C for 1.5 hours. After washing with 10 bed volumes of lysis buffer containing 0.9 mM Tween 20, SK1 was eluted using buffer with 250 mM imidazole and 0.9 mM Tween 20.

Cysteine mutants were labeled with two rounds of a 10-fold molar excess of 1-oxyl-2,2,5,5-tetramethylpyrroline-3-methyl methanethiosulfonate (Enzo Life Sciences) per cysteine on ice in the dark over a 2-hour period. The samples were then incubated on ice at 4 °C overnight (~15 hours) to yield the spin-labeled side chain R1. Unreacted spin label was removed by size-exclusion chromatography using a Superdex 200 Increase 10/300 GL column (GE Healthcare) equilibrated in 50 mM Tris·HCl, pH 7.8, 200 mM NaCl, and 10% (vol/vol) glycerol, with or without 0.9 mM Tween 20. Peak fractions of purified SK1 were pooled and concentrated using an Amicon Ultra 10,000 MWCO filter concentrator (Millipore), and the final protein concentration was determined by A280 measurement (ε = 49,390 M^−1^·cm^−1^) for use in subsequent studies.

### Fluorescence-based sphingosine kinase assay

The functional integrity of spin-labeled DEER mutants was evaluated using a fluorescence-based sphingosine kinase assay, as previously described, with 15-NBD-sphingosine serving as the substrate^38^. Reactions were carried out in a buffer containing 50 mM HEPES (pH 7.4), 15 mM MgCl_2_, 10 mM KCl, 0.005% Triton X-100, and 10% glycerol. The reaction mixture consisted of 20 μM NBD-sphingosine and 1 mM ATP in a final volume of 100 μL. Reactions were initiated by the addition of 1 μM of each SK1 mutant and incubated at 37 °C for 30 minutes. Reactions were quenched by the addition of 100 μL of 1 M potassium phosphate buffer (pH 8.5), followed by extraction with 500 μL of chloroform/methanol (2:1). After centrifugation at 15,000 rpm for 1 minute, 100 μL of the upper aqueous layer was transferred to a 96-well microplate and mixed with 100 μL of dimethylformamide. Fluorescence was measured on a SpectraMax i3 microplate reader with excitation at 485 nm and emission at 538 nm. A reaction lacking enzyme served as a blank control. Each assay was performed in triplicate (technical replicates). For each mutant, the amount of generated NBD-S1P was calculated and normalized to the value obtained for cysteine-less SK1.

### ATPase activity assay

ATPase activity was measured using the malachite green method, as previously described, with Biomol^®^ Green reagent used to detect the release of free inorganic phosphate (P_i_)^39^. Reactions were carried out in a buffer containing 50 mM MOPS (pH 7.4), 10 mM NaCl, and 10 mM MgCl_2_. ATP (100 μM) was added to 50 μL of reaction mixture and transferred to a 96-well plate. Reactions were initiated by adding an equal volume of each SK1 mutant (prepared in the same buffer), resulting in a final SK1 concentration of 0.5 μM per well. Plates were incubated at room temperature for 1 hour. Each SK1 mutant was tested in triplicate. Control reactions lacking ATP were included to account for background signal. Reactions were terminated by adding 200 μL of Biomol^®^ Green reagent per well, followed by incubation at room temperature for 30 minutes to allow color development. Absorbance was measured at 620 nm using a SpectraMax i3 microplate reader. For each mutant, the amount of released phosphate was calculated from a standard curve and normalized to the value obtained for cysteine-less SK1.

### SK1-liposome sample preparation

Cholesterol, 1-palmitoyl-2-oleoyl-sn-glycero-3-phosphocholine (POPC), 1-palmitoyl-2-oleoyl-sn-glycero-3-phosphoethanolamine (POPE), 1-palmitoyl-2-oleoyl-sn-glycero-3-phospho-L-serine (POPS), and L-α-phosphatidylinositol (Liver, Bovine, SAPI) (Avanti Polar Lipids) were combined in a 20:14:35:22:9 molar ratio, dissolved in chloroform, evaporated to dryness using a rotary evaporator, and desiccated overnight under vacuum in the dark. The dried lipids were rehydrated in 50 mM Tris·HCl (pH 7.5), 100 mM NaCl, and 10% glycerol buffer to a final concentration of 40 mM, followed by homogenization through 10 freeze-thaw cycles. The resulting lipid suspension was aliquoted and stored at −80 °C. Large unilamellar vesicles (LUVs) were prepared by sequential extrusion of the homogenized lipids through polycarbonate membranes (Avanti) with pore sizes of 0.4, 0.2, and 0.1 μm, performing ≥10 passes through each membrane. SK1 mutants were mixed with liposomes at a 1:1500 molar ratio and concentrated using a 10,000 MWCO filter concentrator to increase total spin concentration.

### CW-EPR and DEER spectroscopy

CW-EPR spectra of spin-labeled SK1 samples were collected at room temperature on a Bruker EMX spectrometer operating at X-band frequency (9.5 GHz) using 10-mW incident power and a modulation amplitude of 1.6 G. DEER spectroscopy was performed on an Elexsys E580 EPR spectrometer operating at Q-band frequency (33.9 GHz) with the dead-time free four-pulse sequence at 83 K^41^. Pulse lengths were 20 ns (π/2) and 40 ns (π) for the probe pulses and 40 ns for the pump pulse. The frequency separation was 63 MHz. Ligands were added in excess relative to the protein, resulting in final concentrations of 10 mM ATP, 10 mM MgSO_4_, and 0.55 mM sphingosine, FTY720, SKI-II, or PF-543. The sample pH was adjusted to 7.4 and verified using a pH microelectrode. For DEER analysis, samples were cryoprotected with 24% (vol/vol) glycerol and flash-frozen in liquid nitrogen.

Primary DEER decays were analyzed using a home-written software (DeerA, Dr. Richard Stein, Vanderbilt University) operating in the Matlab (MathWorks) environment as previously described^42^. Briefly, the software carries out global analysis of the DEER decays obtained under different conditions for the same spin-labeled pair. The distance distribution is assumed to consist of a sum of Gaussians, the number and population of which are determined based on a statistical criterion. The generated confidence bands were determined from calculated uncertainties of the fit parameters. We also analyzed DEER decays individually and found that the resulting distributions agree with those obtained from global analysis. Comparison of the experimental distance distributions with the crystal structures using a rotamer library approach was facilitated by the MMM 2018.2 software package^43^. Rotamer library calculations were conducted at 175 K.

### Molecular Dynamics simulations

Four systems were prepared for molecular dynamics (MD) simulations: (1) Apo, (2) Apo with Ser225 phosphorylated, (3) Mg^2+^ATP/Sph-bound SK1, and (4) Mg^2+^ATP/Sph-bound SK1 with phosphorylated Ser225. Each system was simulated in triplicate, with each replicate run for 500 ns. For the non-phosphorylated systems, the crystal structure of SK1 (PDB code 3VZB) was used as the starting model^15^. For the phosphorylated systems, an AlphaFold 3-predicted model was employed^32^. Histidine residues H156 and H355 were protonated using CHARMM-GUI^44^, based on pKa predictions obtained from PROPKA, with the pH set to 7.4 to mimic physiological conditions. All systems were prepared using the CHARMM-GUI Membrane Builder, which positioned the protein at the membrane interface of a heterogeneous lipid bilayer composed of cholesterol, POPC, POPE, POPS, and SAPI lipids at a molar ratio of 20:14:35:22:9. Membrane positioning was optimized using PPM 2.0^45^. Each protein–membrane complex was solvated in explicit TIP3P water and neutralized with Na+ and Cl− ions. An additional concentration of 0.15 M NaCl was added. The final system contained approximately 218,000 atoms.

Simulations were performed with the CHARMM36m all-atom additive force field^46^ using the NAMD 3 simulation engine^47^. Initial energy minimization was conducted for 10,000 steps using conjugate gradient technique. The systems were then equilibrated for approximately 19 ns prior to production runs using a 6-step restraining regimen which was performed in an NVT ensemble based on the CHARMM-GUI procedures for protein simulations^48^. Production MD simulations were run in an NPT ensemble with a 2-fs time step at 310 K using a Langevin integrator with a damping coefficient of 1.0 ps^−1^. Pressure was maintained at 1 atm using the Nose-Hoover Langevin piston method^49,50^. RMSD analyses were carried out with reference to the first frame of the selected trajectory. Salt bridge analysis was based on the minimum distance calculated between donor and acceptor atoms, with a cutoff distance of 4.5 Å used to define a salt bridge. Results were visualized by plotting the minimum distance as a function of simulation time. For lipid contact analysis, a cutoff distance of 4.0 Å was used to define contacts between the protein and lipid molecules.

## Extended Data

## Data Availability

The generated data, including those from the DEER experiments, are available in the manuscript or the supplementary materials. Source data including DEER, MD trajectories, and DeerA software have been deposited to the Zenodo repository maintained by CERN, https://doi.org/xxxxx. Other data that support this study are available from the corresponding authors upon reasonable request.
